# Prenatal adverse environment is associated with epigenetic age deceleration at birth and hypomethylation at the hypoxia-responsive *EP300* gene

**DOI:** 10.1186/s13148-019-0674-5

**Published:** 2019-05-09

**Authors:** Helena Palma-Gudiel, Elisenda Eixarch, Fátima Crispi, Sebastián Morán, Anthony S. Zannas, Lourdes Fañanás

**Affiliations:** 1 Department of Evolutionary Biology, Ecology and Environmental Sciences, Faculty of Biology, University of Barcelona (UB), Avda. Diagonal 643 2n A, 08028 Barcelona, Spain; 2 Centro de Investigación Biomédica en Red en Salud Mental (CIBERSAM), Madrid, Spain; 3 Fetal i+D Fetal Medicine Research Center, BCNatal–Barcelona Center for Maternal-Fetal and Neonatal Medicine (Hospital Clínic and Hospital Sant Joan de Déu), Institut Clínic de Ginecologia, Obstetrícia i Neonatologia, Institut d’Investigacions Biomèdiques August Pi i Sunyer, Universitat de Barcelona, Barcelona, Spain; 4 Center for Biomedical Research on Rare Diseases (CIBER-ER), Madrid, Spain; 5 Cancer Epigenetics and Biology Program (PEBC), Bellvitge Biomedical Research Institute (IDIBELL), L’Hospitalet de Llobregat, Barcelona, Spain; 6 Departments of Psychiatry and Genetics, University of North Carolina, Chapel Hill, NC USA

**Keywords:** DNA methylation, Obstetric complications, Prenatal stress, Hypoxia, EP300 gene, Epigenetic clock, Monozygotic twins, Schizophrenia

## Abstract

**Background:**

Obstetric complications have long been retrospectively associated with a wide range of short- and long-term health consequences, including neurodevelopmental alterations such as those observed in schizophrenia and other psychiatric disorders. However, prospective studies assessing fetal well-being during pregnancy tend to focus on perinatal complications as the final outcome of interest, while there is a scarcity of postnatal follow-up studies. In this study, the cerebroplacental ratio (CPR), a hemodynamic parameter reflecting fetal adaptation to hypoxic conditions, was analyzed in a sample of monozygotic monochorionic twins (60 subjects), part of them with prenatal complications, with regard to (i) epigenetic age acceleration, and (ii) DNA methylation at genes included in the polygenic risk score (PRS) for schizophrenia, and highly expressed in placental tissue.

**Results:**

Decreased CPR measured during the third trimester was associated with epigenetic age deceleration (β = 0.21, *t* = 3.362, *p* = 0.002). Exploration of DNA methylation at placentally expressed genes of the PRS for schizophrenia revealed methylation at cg06793497 (*EP300* gene) to be associated with CPR (β = 0.021, *t* = 4.385; *p* = 0.00008, FDR-adjusted *p* = 0.11). This association was reinforced by means of an intrapair analysis in monozygotic twins discordant for prenatal suffering (β = 0.027, *t* = 3.924, *p* = 0.001).

**Conclusions:**

Prenatal adverse environment during the third trimester of pregnancy is associated with both (i) developmental immaturity in terms of epigenetic age, and (ii) decreased CpG-specific methylation in a gene involved in hypoxia response and schizophrenia genetic liability.

**Electronic supplementary material:**

The online version of this article (10.1186/s13148-019-0674-5) contains supplementary material, which is available to authorized users.

## Main text

### Background

Prenatal environment constitutes the first modulating agent the developing fetus encounters as it progresses through gestation. The tremendous impact of any environmental threat occurring during this period for both short- and long-term consequences is now widely accepted and well-known as the Developmental Origins of Health and Disease (DOHaD) hypothesis [1]. Also known as the theory of fetal programming, the embedding of early life and its ability to exert long-term effects in late-life is thought to rely on epigenetic mechanisms [2, 3].

Recently, several DNA methylation-based epigenetic clocks have been developed in order to predict chronological age with high accuracy [4, 5]; afterward, Knight and colleagues developed a new predictor specifically aimed to predict gestational age (GA) in perinatal samples [6]. Although epigenetic and chronological age robustly show high correlation across studies, the difference between both variables allows the estimation of the so-called age acceleration (i.e., when epigenetic age is higher than chronological age).

On the one hand, epigenetic age acceleration in adult subjects has been associated with cumulative lifetime stress, lifestyle, and all-cause mortality, among others, suggesting its utility as a better predictor for life expectancy than chronological age itself [7–9]. On the other hand, epigenetic GA deceleration (i.e., when chronological age is higher than epigenetic age), as measured in cord blood, has been described in newborns born to women with low socioeconomic status, Sjögren syndrome, insulin-treated gestational diabetes mellitus, and experiencing antenatal depressive symptoms [6, 10, 11]. Such findings suggest that newborns exposed to prenatal stressors are born in an immature state independently of their chronological GA. In this regard, boys—but not girls—who exhibited lower epigenetic GA at birth exhibited more internalizing problems, such as anxious-depressive symptoms or somatic complaints, at follow-up (mean age 3.7 years), suggesting they are born with a developmental disadvantage [11].

Nevertheless, there is a dearth of studies examining the putative relationship between ultrasound parameters acquired during pregnancy and epigenetic GA acceleration. In this regard, the cerebroplacental ratio (CPR) has been reported to be associated with adverse perinatal outcomes not only in growth-restricted fetuses, but also in low-risk population [12, 13]. Briefly, CPR is calculated by dividing the middle cerebral artery (MCA) pulsatility index (PI) by the umbilical artery (UA) PI [14]. The PI is a parameter reflecting vascular impedance or resistance, i.e., decreased blood flow. Specifically, fetal brain blood supply is known to increase in front of hypoxic stimuli thus decreasing PI in the MCA [15]; while placental insufficiency decreases umbilical blood flow hence increasing UA PI, and has been associated with both short- and long-term detrimental outcomes, including increased cardiovascular risk and deficits in cognition [16, 17]. Consequently, a decreased CPR reflects the combination of both alterations and is an indicator of fetal adaptation to adverse conditions [12].

Obstetric complications (OCs) constitute one of the risk factors more reliably associated with psychopathology, particularly with neurodevelopmental disorders; specifically, the putative association between OCs and schizophrenia has been debated since the 1970s [18–20]. In this regard, a recent umbrella review evaluating all published meta-analysis regarding risk factors and biomarkers for schizophrenia spectrum disorders revealed a history of OCs to significantly increase the risk for developing the disorder with an odds ratio of 2 [21]. Furthermore, exposure to severe OCs together with increased genetic vulnerability, as measured with the polygenic risk score (PRS) for schizophrenia, interact to increase the risk to suffer the disorder up to an odds ratio of 8.36 [22]. In the same study, authors further explored the putative relevance of placental expression of genes included in the PRS; following this approach, they reported (i) an enrichment of PRS genes expressed in placental tissue and (ii) differential expression of PRS genes in placentae from complicated pregnancies (specifically in pre-eclampsia and intrauterine growth restriction). Specifically, the described gene-environment interaction between exposure to OCs and the PRS for schizophrenia was driven by those genes highly expressed in placenta and/or dynamically regulated in complicated pregnancies [22]. Since CPR is a robust indicator of prenatal stress and a predictor of perinatal and long-term morbidity, DNA methylation analysis of genes included in the placental PRS for schizophrenia could shed light on the epigenetic mechanisms mediating the interaction between OCs and neurodevelopmental disorders.

Monozygotic twins have been instrumental for the elucidation of environmental and genetic risks in the etiology of complex traits and disorders. Actually, the differential role of the prenatal environment in shaping psychopathological proneness was first described thanks to monozygotic twin designs [23–25]; these pioneering studies focused on dermatoglyphic measures assessed at birth, which can be used as surrogate measures of altered neurodevelopment during the second trimester of pregnancy [26]. Furthermore, monozygotic twin pregnancies and, more specifically, monochorionic twin pregnancies—i.e., those in which both fetuses share the placenta—are at a higher risk of obstetric complications, the more prevalent being twin-to-twin transfusion syndrome (TTTS) and selective intrauterine growth restriction (sIUGR) [27–29]. Thus, the thorough and prospective ultrasound assessment of prenatal development through monochorionic twin pregnancies offers a quasi-experimental study design in which the genetic and environmental components of epigenetic variability can be dissected.

The objective of the current study was to investigate whether prenatal adverse environment (i) alters human development in terms of epigenetic age, and if (ii) it can get embedded through epigenetic mechanisms in genes previously identified as risk factors for schizophrenia acting during prenatal stages. We hypothesized that a higher exposure to prenatal adverse environment would be associated with (i) delayed development and (ii) DNA methylation at genes involved in the pathogenesis of schizophrenia. While CPR can have diverse effects on genome-wide DNA methylation, with potential relevance for a multitude of phenotypes, the present study a priori examined how CPR epigenetically regulates risk genes for schizophrenia, previously described to interact with the presence of OCs [22].

### Results

#### GA estimation using Knight’s epigenetic clock

**Fig. 1:**
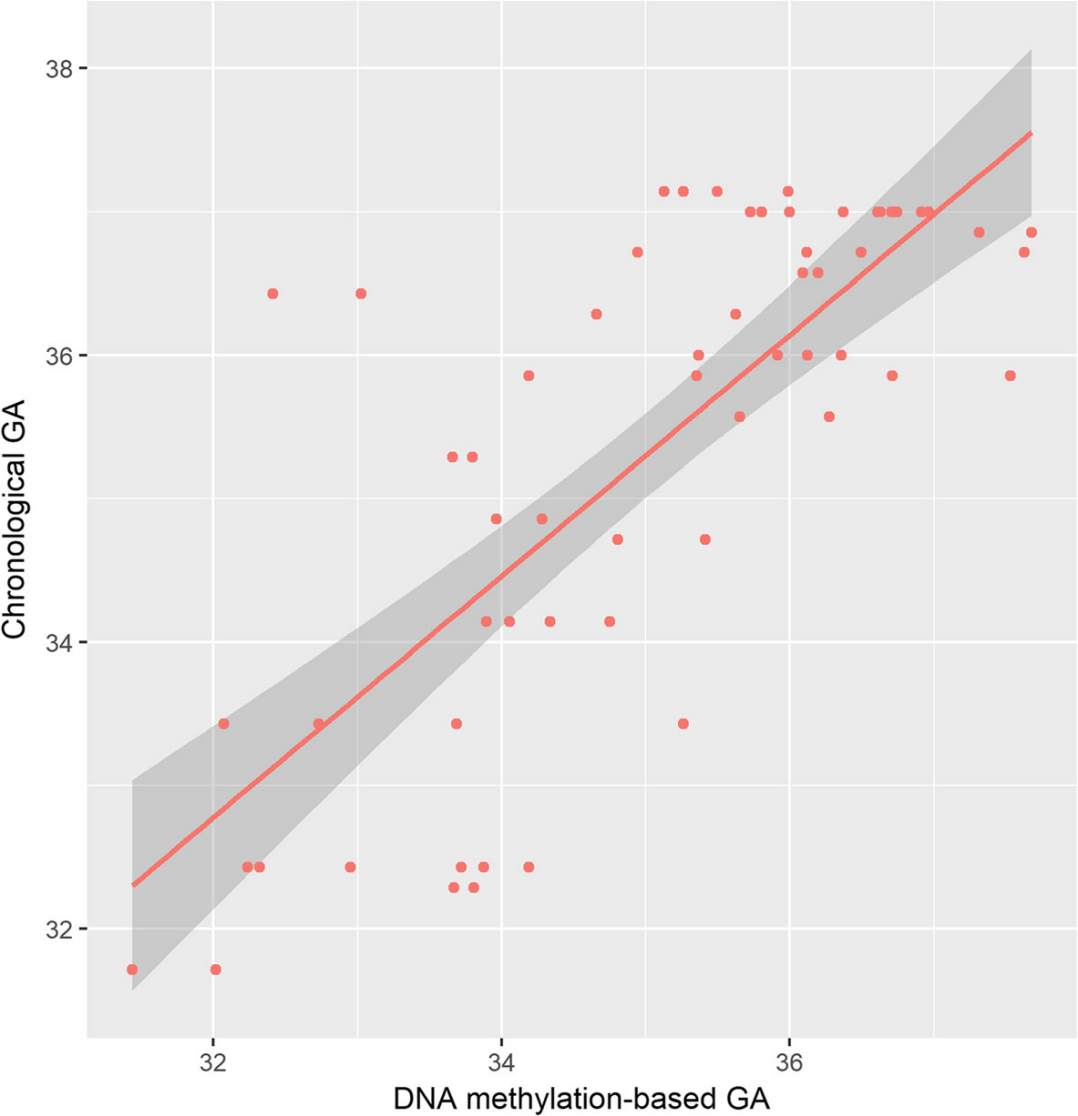
Correlation between chronological GA and epigenetic GA. Chronological GA was calculated using first-trimester crown-rump length measurement of the larger twin, and epigenetic age was calculated based on DNA methylation-based Knight’s clock. Both GA estimations were significantly correlated (*r* = 0.76; *p* = 1.68 × 10^−12^)

After exclusion of two twin pairs (see Methods section), the final sample size was 30 twin pairs. The mean GA at birth of our twin cohort (*n* = 30 twin pairs) was 35.3 weeks (range = 31.7–37.1) and the mean DNA methylation GA at birth was 35 weeks (range = 31.4–37.7). To validate the epigenetic clock predictor in our sample, DNA methylation-based GA was tested for correlation with chronological GA (*r* = 0.76, *p* = 1.68 × 10^−12^; Fig. 1). The average absolute difference between epigenetic GA and chronological GA—hereinafter referred as ΔGA—was 0.9 weeks (range = 0.03–4.02), i.e., 6.3 days. In agreement with previous studies, there was a significant negative correlation between ΔGA and chronological GA (*r* = − 0.47; *p* < 0.001).

#### Association between ΔGA and CPR

**Fig. 2:**
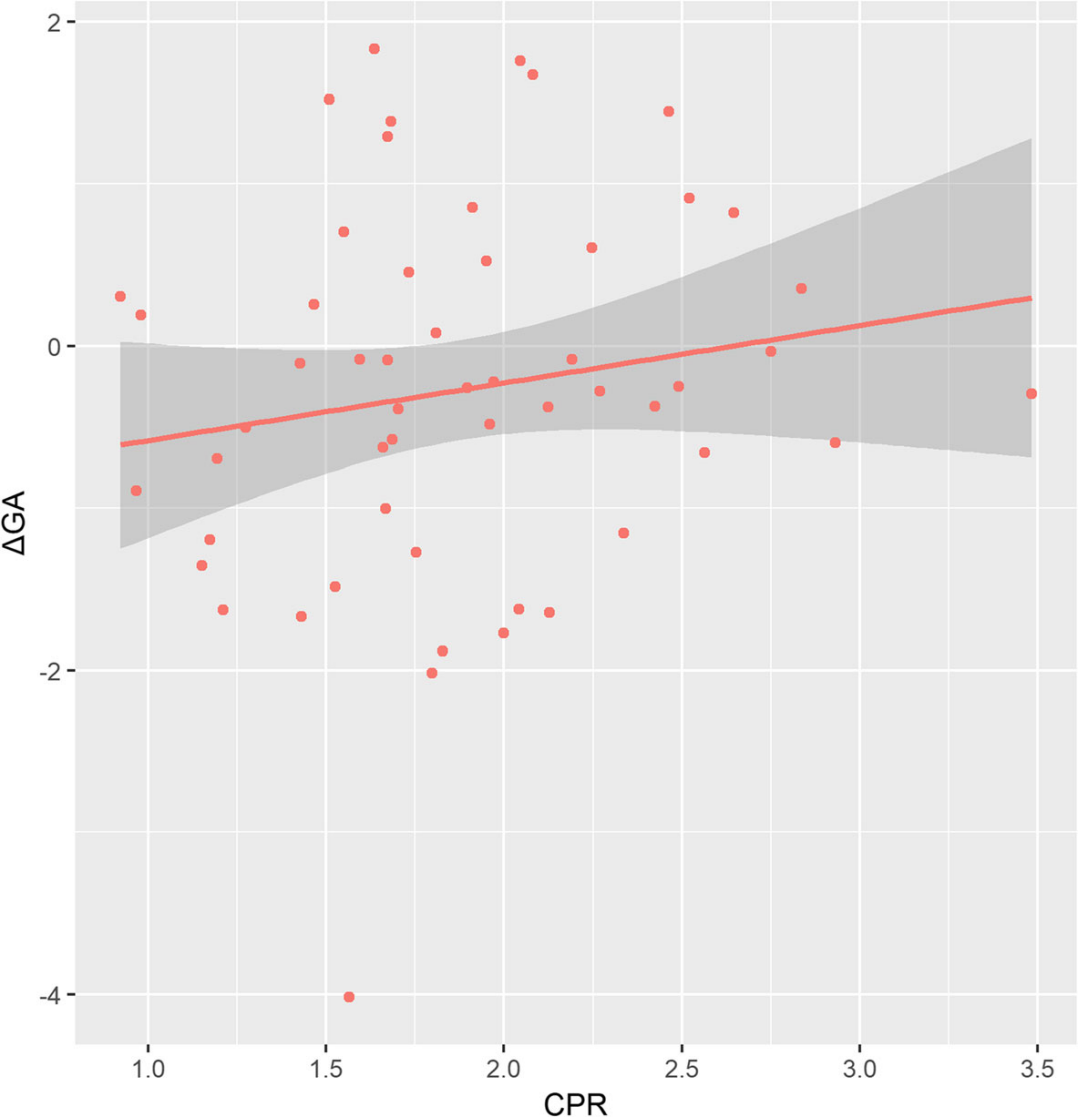
Association between epigenetic age acceleration and cerebroplacental ratio measured during the third trimester. Epigenetic age delta (ΔGA) corresponds to estimated epigenetic age minus chronological age. Thus, ΔGA-positive values reflect epigenetic age acceleration while negative values point out the presence of epigenetic age deceleration. The cerebroplacental ratio (CPR) is calculated as the ratio between the MCAPI and UAPI. Both variables were significantly correlated when adjusting for sex, chronological gestational age, birth weight, and gestational age at ultrasound

ΔGA was tested for associations with CPR measured during the third trimester (mean = 33.8 weeks, range = 28.3–36.4), a few days before childbirth (median = 6.5 days). CPR was significantly associated with ΔGA (β = 0.21, *t* = 3.362, *p* = 0.002) when adjusting for sex, birthweight, diagnostic of either TTTS or sIUGR, surgery time interval (when laser fetoscopy had been applied), and gestational age at ultrasound as covariates. The positive association between CPR and ΔGA remained significant after correction for cell type proportion (β = 0.21, *t* = 2.616, *p* = 0.01). Figure 2 shows the positive association between third trimester CPR and ΔGA.

#### Epigenetic exploration of placental PRS for schizophrenia with regard to CPR

**Fig. 3:**
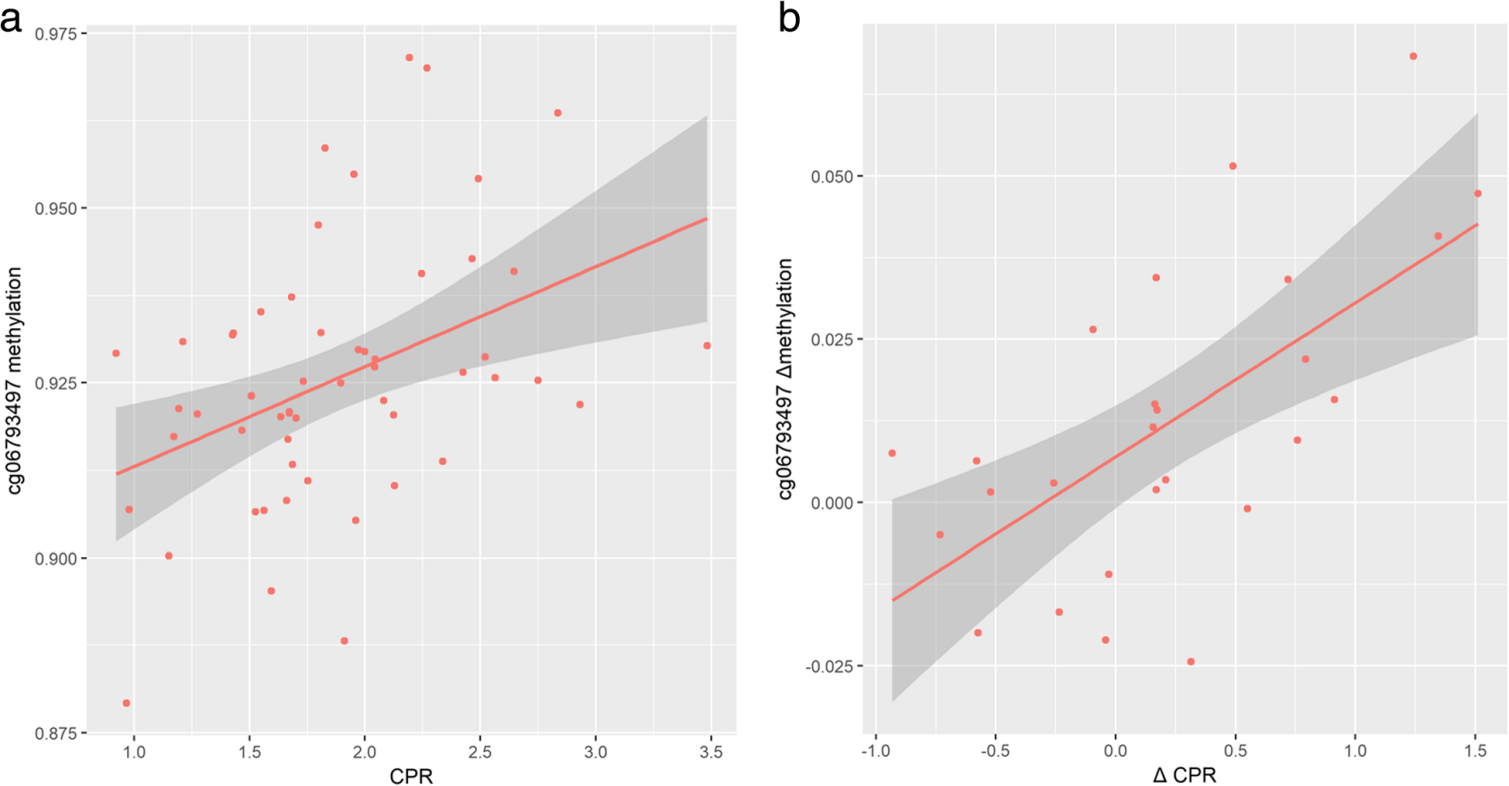
Schizophrenia PRS methylation exploration with regard to cerebroplacental ratio measured during the third trimester. **a** Methylation at cg06793497 (*EP30*0 gene) was significantly associated with CPR in the whole sample (*n* = 54 twin subjects). **b** Intrapair methylation difference at cg06793497 was significantly associated with intrapair CPR difference (*n* = 26 twin pairs)

Following the approach developed by Ursini and collaborators (2018), association between CPR and DNA methylation was tested in all CpG sites included in the DNA methylation array located within genes of the PRS for schizophrenia expressed in placental tissue (placental PRS) [22]. There were 1400 CpG sites annotated to placental PRS genes out of 866,091 CpG sites included in the array. After FDR correction for multiple testing, methylation at one single CpG site, cg06793497, was significantly associated with CPR (β = 0.021, *p* = 0.00008, *t* = 4.385; *q*_FDR adjusted_ = 0.11; Fig. 3a), such that increased cg06793497 methylation was associated with increased CPR. The top 10 CpG sites yielded by this approach are summarized in Table 1 (all *q* values > 0.75).

**Table 1:** Top 10 CpG sites of the PRS methylomic exploration in association with CPR (1400 CpG sites tested)

#	CpG probe^a^	Genomic coordinates^b^	Gene	PRS exploration^c^			Intrapair^d^	
				beta	*p* value	q value	beta	*p* value
1	cg06793497	22: 41,542,898	*EP300*	0.021	8.2E-05	0.115	0.027	0.001
2	cg15620905	1: 44,024,150	*PTPRF*	0.040	0.002	0.804	0.008	0.563
3	cg12252443	2: 198,364,630	*HSPD1*	-0.006	0.002	0.804	− 0.007	0.055
4	cg24936500	2: 233,499,637	*EFHD1*	0.022	0.004	0.804	0.001	0.849
5	cg00262246	3: 136,007,461	*PCCB*	0.012	0.004	0.804	0.012	0.029
6	cg22495590	5: 138,161,059	*CTNNA1*	-0.020	0.004	0.804	− 0.010	0.203
7	cg14902598	5: 138,210,650	*CTNNA1*	0.017	0.005	0.804	0.006	0.371
8	cg01024069	14: 104,158,878	*KLC1*	-0.010	0.005	0.804	− 0.010	0.033
9	cg16362480	16: 30,077,084	*ALDOA*	0.031	0.005	0.804	0.024	0.126
10	cg12955069	16: 58,593,852	*CNOT1*; *SNORA50*	-0.016	0.006	0.804	− 0.021	0.026

^a^ CpG site code according to the Illumina annotation

^b^Genomic coordinates correspond to hg19 built

^c^Refers to the statistics of each analysis in the first model encompassing 1400 CpG sites located in genes of the PRS for schizophrenia highly expressed in the placenta as described by Ursini et al.

^d^Refers to intrapair comparison of DNA methylation values and CPR. Thus, the *n* for these analyses was of 26 twin pairs

To further explore the association between cg06793497 methylation and CPR, it was analyzed in a monozygotic twin intrapair design. The intrapair twin design further allows controlling for chronological GA, sex, and timing of the Doppler ultrasound, since these variables are shared by co-twins of a pair. Four observations were removed from the analysis due to missingness for any of the variables in one of the co-twins of a pair. Thus, intrapair differences for these measures were calculated for all twin pairs of the sample were both measures were available for both twins of a pair (*n* = 27 twin pairs). Intrapair differences in cg06793497 methylation and CPR, measured during the third trimester, were significantly correlated (*r* = 0.64, *p* < 0.001; Fig. 3b). The association between both variables remained significant after adjusting for cell type count intrapair differences (β = 0.027, *t* = 3.924, *p* = 0.001). Intrapair exploration of the top 10 CpG sites (Table 1) revealed significant associations between CPR and DNA methylation at CpG probes cg00262246 (β = 0.012, *p* = 0.029), cg01024069 (β = − 0.01, *p* = 0.033), and cg12955069 (β = − 0.021, *p* = 0.026).

#### DNA methylation exploration of *EP300* gene

To further explore the putative relevance of DNA methylation at other CpG sites located within the *EP300* gene and its surrounding regions, DNA methylation at 27 CpG sites included in the array and annotated to this region was also explored with regard to CPR (see Table 2). All analyses were adjusted for cell sex, birthweight, gestational age at ultrasound, and cell type count. In addition to cg06793497, two additional CpG sites—cg12968540 and cg19011939—were significantly associated with CPR (*p* < 0.05); moreover, methylation at four additional CpG sites—cg04452260, cg24349919, cg11931284 and cg25888227—showed trend associations with CPR (*p* < 0.01). The intrapair approach was then applied for these newly identified six CpG sites revealing cg11931284 (β = 0.028, *t* = 2.985, *p* = 0.008) and cg19011939 (β = − 0.021, *t* = − 2.343, *p* = 0.03) to be significantly associated with CPR, when adjusting for cell types intrapair differences.

**Table 2:** List of CpG sites included in the array located in the *EP300* gene and its surrounding CpG island and antisense ncRNA (*EP300-AS1*)

CpG probe	Genomic coordinates	beta	*p* value
cg00500400	41487283	0.001	0.67
cg04452260	41487569	0.003	0.09
cg09331127	41487734	− 0.0006	0.79
cg02046995	41487740	0.004	0.49
cg24349919	41487761	0.005	0.09
cg02107564	41488750	− 0.005	0.48
cg03427564	41489051	0.004	0.21
cg03656483	41490340	− 0.001	0.65
cg00187244	41492007	0.011	0.23
cg11931284	*41492370*	*0.015*	*0.07*
cg13028324	41501680	− 0.0005	0.89
cg20730595	41513219	− 0.001	0.85
cg17439569	41513539	− 0.005	0.16
cg05997318	41542772	− 0.014	0.12
cg06793497	*41542898*	*0.021*	*0.00008*
cg06329185	41544246	− 0.0008	0.75
cg07345240	41556691	0.002	0.73
cg25299898	41563501	0.003	0.74
cg12968540	41572924	0.009	0.008
cg26901641	41573032	− 0.006	0.12
cg03950371	41573046	− 0.003	0.48
cg14455139	41573155	− 0.0001	0.98
cg05601844	41573176	− 0.0005	0.89
cg19011939	*41591607*	− *0.01*	*0.01*
cg12917725	41592634	0.002	0.76
cg25888227	41593581	0.004	0.07
cg22037654	41593650	0.0001	0.94

DNA methylation at CpG sites highlighted in italics was significantly associated with CPR measured during the third trimester in an intrapair approach

### Discussion

To the best of our knowledge, this is the first study analyzing the epigenetic age in association with adverse prenatal environment as measured by a hemodynamic ultrasound parameter. Firstly, we describe the significant association between CPR measured during the third trimester of pregnancy with epigenetic age acceleration. Specifically, subjects exhibiting decreased CPR—exposed to prenatal adverse conditions—were born with decelerated epigenetic age, i.e., prenatally stressed subjects were born immature adjusting for their gestational age at birth. Additionally, methylomic exploration of schizophrenia PRS genes known to be expressed in placenta revealed the association between CPR and *EP300* gene CpG-specific methylation, at the cg06793497 probe, in our monochorionic twin sample.

Developmental deficits and developmental delays have been previously described in children who would later develop schizophrenia [30]; although such prodromal symptoms were in accordance with the neurodevelopmental hypothesis for schizophrenia, biological mechanisms mediating these effects remain largely unknown. Epigenetic immaturity in response to prenatal stress could be contributing to this developmental delay. Interestingly, epigenetic age deceleration has been previously described in association with maternal pathologies during pregnancy, such as maternal depression or Sjögren’s syndrome, suggesting it can be a robust biomarker of prenatal suffering [10, 11]. It is worth noting that CPR was measured a few days prior to childbirth; thus, it can be used as a surrogate marker of prenatal adaptation to adverse conditions experienced at the end of the pregnancy, i.e., as a marker of perinatal risk.

Integration of the schizophrenia PRS [31] with obstetric and placental information [22], allowed the identification of *E1A binding protein p300* (*EP300*) gene CpG-specific methylation as a putative marker of exposure to prenatal stress. Interestingly, the *EP300* gene encodes a histone acetyltransferase (HAT) involved in several cell pathways such as cell proliferation and differentiation. Mutations at *EP300* gene have been described to cause Rubinstein-Taybi syndrome, a rare autosomal dominant neurodevelopmental disorder characterized by intellectual disability, psychomotor and language delay, and facial dysmorphisms [32]. Likewise, these symptoms, including developmental delay, learning problems, and cleft palate, characterize the 22q11.2 deletion syndrome, a well-defined congenital condition caused by the deletion of the 22q11.2 segment [33]. Notably, this syndrome is associated with a higher risk to develop schizophrenia, among other psychiatric conditions [34]; interestingly, *EP300* gene is located on chromosome 22 at position 22q13.2.

Further exploration of differential DNA methylation in and around the EP300 gene revealed cg19011939 to be differentially methylated in association with prenatal adversity. While higher exposure to a prenatal adverse environment, as reflected by lower CPR during the third trimester, is associated with decreased methylation at cg06793497 in the hypoxia-responsive *EP300* gene, there appears to be increased methylation at cg19011039 at *EP300-AS1* gene. Thus, we speculate that higher exposure to prenatal stress might be associated with reciprocal patterns of *EP300* and *EP300-AS1* epigenetic regulation that could act synergistically, a hypothesis that may be explored in future studies [35].

Remarkably, *EP300* has been identified as a co-activator of the hypoxia-inducible factor 1 alpha (*HIF1A*). In this regard, hypoxic conditions stimulate *EP300* expression, which has a neuroprotective role [36]. Accordingly, genetic variability at *EP300* gene has been associated with human adaptations to high altitude regions, e.g., the Tibet [37]. Likewise, pre- and peri-natal hypoxia have been associated with schizophrenia spectrum disorders, particularly by decreasing hippocampal volume [38, 39]; complementarily, a decreased or impaired response to hypoxia via neurotrophic factors has also been implicated in the etiology of schizophrenia [40]. Furthermore, DNA methylation at the *IGF2BP1* gene, also involved in prenatal development [41], has been associated with both adult working memory and birthweight [42]; further highlighting the advantage of twin study designs to identify environmentally-driven epigenetic consequences of prenatal stress. Overall, these findings point to the existence of a GxE interaction between genetic vulnerability and exposure to prenatal hypoxia, as already highlighted by Ursini and collaborators [22]. In this framework, *EP300* methylation could be one of the mediators of such interaction.

A number of limitations of the present study should be noted. First, the moderate sample size (*n* = 60 subjects, 30 twin pairs) limits the statistical power of the analysis; however, smaller sample sizes (*n* = 22 MZ twin pairs) have been described to be sufficient to identify methylation differences of 6% with > 80% power [43]. Moreover, a lenient significance threshold after correction multiple testing was used; however, previous epigenetic studies have described FDR values between 5 and 20% as markers of medium-confidence sites [44]. Another limitation regards the moderately small reported effect sizes (around 2%) questioning the biological relevance of our findings [45]; however, these findings are in agreement with a larger body of evidence regarding cord blood methylation after exposure to a number of prenatal stressors. Such small DNA methylation changes may act in conjunction with a myriad of other epigenetic signatures and biological processes in order to maintain homeostasis in the face of threats. Additionally, although epigenomic information was available from a methylomic array including more than 800,000 CpG sites distributed throughout the whole human genome, only 1400 CpG sites were analyzed; alternative approaches including the total of CpG sites included in the array would have yield different findings, probably pointing to genes involved in other neurodevelopmental disorders besides schizophrenia. Furthermore, while the set of genes analyzed in the current approach were described to be highly expressed in placental tissue [22], placentae were not available for this sample and cord blood was thus analyzed as the proxy tissue of choice with regard to exposure to prenatal adversity. Finally, MZ twin pregnancies are characterized by lower gestational ages at birth than singleton pregnancies; besides, obstetric scales commonly used in psychiatric studies include twin pregnancies as an obstetric complication. Thus, findings derived from the present design might not be generalizable to the general population.

### Conclusions

Further studies are needed to test the time stability of the hereby identified methylation signature. It will be equally relevant to explore neurobehavioral correlates of EP300 methylation during early childhood along with its putative association with neurodevelopmental outcomes, including psychosis liability. Additionally, a longitudinal follow-up is required to test the role of postnatal environment in these phenotypes since both epigenetic age deceleration and CpG-specific differential methylation in association with CPR could return to basal levels after birth. Finally, genetic exploration of these subjects regarding schizophrenia PRS will be instrumental for the study of GxE interactions and genetic liability for an impaired hypoxia response during human development.

### Methods

#### Study population

This was a prospective study including fetal pairs from monochorionic diamniotic twin pregnancies attended at Hospital Clínic de Barcelona (Spain) during a 2-year recruitment period. Monochorionic monoamniotic twin pregnancies were excluded from the present study to avoid putative confounding with regard to differential exposure to stress in both types of twin pregnancies. The study protocol was approved by the hospital ethics committee (HCB/2016/0046), and all patients provided written informed consent.

We included 32 monochorionic pregnancies (*n* = 64 samples). The sample was enriched for two monochorionic-specific severe obstetric complications: twin-to-twin transfusion syndrome (TTTS, *n* = 8) and selective intrauterine growth restriction (sIUGR, *n* = 9). All TTTS cases were treated upon detection by means of laser fetoscopy [46].

Maternal age and pre-pregnancy BMI were retrieved from hospital records. Gestational age was dated using first-trimester crown-rump length measurement of the larger twin [47].

#### Fetal ultrasound assessment

Ultrasound assessment was performed on a Voluson Expert 8 (General Electrical Medical Systems, Milwaukee, WI, USA) or a Siemens Sonoline Antares (Siemens Medical Systems, Erlangen, Germany) with 8- to 4-MHz or 6- to 4- MHz curved array probes, respectively. All fetuses underwent detailed ultrasound evaluation including fetal anatomy and Doppler measurements such as UAPI, MCAPI and ductus venosus PI. All Doppler evaluations were acquired at a normal fetal heart rate (FHR) in the absence of fetal body or respiratory movements and at an angle of insonation as close to 0° as possible (but always < 15°), and the mechanical and thermal indices were maintained below 1. CPR was calculated as the ratio between MCAPI and UAPI, according to previous studies [12].

#### DNA methylation

Umbilical vein cord blood samples were obtained from the clamped umbilical cord immediately after delivery of the fetus. All blood samples were collected in EDTA-treated tubes and processed within 1 h. Plasma was separated by centrifugation at 3000 rpm for 10 min at 4 °C, and stored at − 80 °C until further use. Genomic DNA was extracted from fetal cord blood using QIAamp DNA Mini Kit (Qiagen). DNA quality and quantity were assessed by NanoDrop One (Thermo Scientific). Genomic DNA was bisulfite converted using the Zymo EZ-96 DNA Methylation Kit (Zymo Research). Genome-wide DNA methylation levels were assessed over 850,000 CpG sites by means of the Infinium MethylationEPIC BeadChip Kit (Illumina Inc., CA, USA) according to the manufacturer’s protocol. Pre-processing and normalization were performed using the Bioconductor minfi package [48]. CpG probes containing common SNPs were discarded. All probes mapping to the X and Y chromosomes were also removed. Finally, cross-hybridizing probes as previously identified were excluded from further analysis [49]. All samples (*n* = 64) were run on the same plate.

Absence of maternal contamination was confirmed after retrieving DNA methylation values at 10 CpG sites previously described to identify sample contamination by maternal blood during sample collection [50]. None of the samples assayed exhibited DNA methylation values above the threshold at 5 or more of those CpG sites (see Additional file 1 for specific methylation values). Two samples (from the same twin-pair) were excluded from further analyses due to lack of monozygosity as assessed by 59 SNPs included in the array. One of the samples was removed from analysis due to insufficient DNA concentration, the co-twin sample was also excluded from further analysis.

#### Statistical analyses

All statistical analyses were conducted in R version 3.5.0 [51]. DNA methylation-based GA prediction was performed using the R code and statistical pipeline developed by Knight, based on the methylation profile of 148 CpG sites [6]; this predictor was developed using 15 Illumina DNA methylation datasets (*n* = 1434 neonates). Following Simpkin et al. recommendations, the Knight clock was preferred for our analysis as it was developed and tested in preterm infants datasets such as our monozygotic twin population, characterized by a mean gestational age at birth of 35.3 weeks [52]. The EPIC array lacks 6 of the CpG sites originally included in the Knight clock, these values were imputed manually as non-available. Interestingly, DNA methylation-based age estimation relying on EPIC array data has already been described to accurately predict age despite the lack of several CpG sites originally included in Horvath’s and Hannum’s clocks [53].

Gestational age acceleration (ΔGA) was calculated as the absolute difference between epigenetic GA and chronological GA. Since ΔGA was associated with chronological GA (*r* = − 0.47; *p* < 0.001), the latter was included as a covariate in all statistical models; this association has been already reported in prior studies exploring epigenetic-based GA estimations at birth [6, 10, 11].

Cell counts of CD4^+^ T cells, CD8^+^ T cells, B cells, NK cells, granulocytes, monocytes, and nucleated red blood cells (nRBCs) were estimated using the R code and statistical pipeline developed by Houseman [54].

A multiple linear regression model was built to analyze the correlation between ΔGA and CPR. Fetal sex, birthweight, diagnostic of either TTTS or sIUGR (binary variable), post-surgery interval (in TTTS cases where laser fetoscopy had been applied), and gestational age at ultrasound were included as independent variables in the model as they are known to influence either DNA methylation (from which ΔGA is calculated) or CPR. This analysis was conducted in the total MZ twin sample (*n* = 60).

DNA methylation at CpG sites annotated to the 43 genes of the Placental PRS1 as described by Ursini et al. [22] was retrieved to test their association with CPR. A second multiple linear regression model was then designed to explore putative effects of CPR upon methylation of PRS genes, testing 1,400 associations. The aforementioned confounding variables along with cell types proportions (CD4^+^ T cells, CD8^+^ T cells, B cells, NK cells, granulocytes, monocytes, and nRBCs) were included as covariates, as they are known to affect methylation values. False discovery rate (FDR) correction for multiple testing was applied, considering *q* values under 20% to be indicative of medium-confidence probes following prior studies [44].

A twin-based approach previously developed in our group [55] was also applied to refine the association between cg06793497 methylation and CPR. Briefly, intrapair differences for both variables of interest were computed for each twin pair; afterward, a regression model was fitted with an estimated intrapair cg06793497 methylation (Δmethylation) and intrapair CPR (ΔCPR). This last model was not adjusted for either sex or chronological gestational age since both variables are identical for both twins of a pair.

## Additional file

Additional file 1: DNA methylation values for CpG probes used to discard the presence of maternal contamination. (DOCX 29.6 kb)
