# Supplementary material for: Prenatal adverse environment is associated with epigenetic age deceleration at birth and hypomethylation at the hypoxia-responsive EP300 gene
Source: Clin Epigenetics. 2019 May 9;11:73. doi: 10.1186/s13148-019-0674-5 (PMC6507133; doi:10.1186/s13148-019-0674-5)
Supplement: Supplementary file 1 — DNA methylation values for CpG probes used to discard the presence of maternal contamination. (DOCX 29.6 kb) [file 13148_2019_674_MOESM1_ESM.docx]

**Additional File**

| **Code** | **cg25556035** | **cg13138089** | **cg12634306** | **cg25241559** | **cg02812891** | **cg15645660** | **cg19509778** | **cg15931839** | **cg16617301** | **cg24767131** |
| --- | --- | --- | --- | --- | --- | --- | --- | --- | --- | --- |
| C01.1 | 0.042 | 0.035 | 0.079 | 0.034 | 0.008 | 0.172 | 0.041 | 0.055 | 0.091 | 0.096 |
| C01.2 | 0.075 | 0.052 | 0.158 | 0.046 | 0.032 | 0.214 | 0.062 | 0.089 | 0.102 | 0.103 |
| C02.1 | 0.046 | 0.033 | 0.106 | 0.022 | 0.006 | 0.178 | 0.081 | 0.091 | 0.105 | 0.094 |
| C02.2 | 0.051 | 0.043 | 0.114 | 0.043 | 0.019 | 0.172 | 0.055 | 0.081 | 0.08 | 0.104 |
| C03.1 | 0.05 | 0.047 | 0.113 | 0.042 | 0.014 | 0.195 | 0.055 | 0.068 | 0.096 | 0.066 |
| C03.2 | 0.042 | 0.031 | 0.112 | 0.041 | 0 | 0.179 | 0.057 | 0.074 | 0.074 | 0.071 |
| C04.1 | 0.049 | 0.03 | 0.107 | 0.031 | 0.02 | 0.156 | 0.003 | 0.084 | 0.102 | 0.062 |
| C04.2 | 0.052 | 0.031 | 0.133 | 0.024 | 0.009 | 0.143 | 0.113 | 0.089 | 0.116 | 0.069 |
| C05.1 | 0.043 | 0.127 | 0.083 | 0.034 | 0.096 | 0.105 | 0.045 | 0.119 | 0.104 | 0.164 |
| C05.2 | 0.045 | 0.048 | 0.107 | 0.027 | 0.016 | 0.104 | 0.073 | 0.115 | 0.103 | 0.129 |
| C06.1 | 0.054 | 0.032 | 0.118 | 0.035 | 0.013 | 0.139 | 0.041 | 0.072 | 0.098 | 0.062 |
| C06.2 | 0.054 | 0.043 | 0.124 | 0.06 | 0.009 | 0.127 | 0.071 | 0.086 | 0.088 | 0.066 |
| C07.1 | 0.056 | 0.065 | 0.139 | 0.057 | 0.019 | 0.159 | 0.094 | 0.089 | 0.09 | 0.117 |
| C07.2 | 0.049 | 0.041 | 0.125 | 0.046 | 0.022 | 0.186 | 0.055 | 0.089 | 0.093 | 0.109 |
| C08.1 | 0.05 | 0.025 | 0.133 | 0.041 | 0.007 | 0.164 | 0.061 | 0.092 | 0.099 | 0.053 |
| C08.2 | 0.052 | 0.029 | 0.143 | 0.04 | 0.014 | 0.161 | 0.066 | 0.085 | 0.098 | 0.076 |
| C09.1 | 0.059 | 0.037 | 0.113 | 0.024 | 0.015 | 0.199 | 0.042 | 0.104 | 0.091 | 0.049 |
| C09.2 | 0.057 | 0.049 | 0.11 | 0.033 | 0.006 | 0.193 | 0.064 | 0.072 | 0.095 | 0.062 |
| C10.1 | 0.025 | 0.032 | 0.125 | 0.031 | 0.011 | 0.169 | 0.092 | 0.093 | 0.097 | 0.063 |
| C10.2 | 0.038 | 0.03 | 0.122 | 0.025 | 0.011 | 0.17 | 0.076 | 0.099 | 0.094 | 0.061 |
| C12.1 | 0.057 | 0.039 | 0.095 | 0.049 | 0.017 | 0.147 | 0.081 | 0.09 | 0.096 | 0.097 |
| C12.2 | 0.085 | 0.078 | 0.146 | 0.038 | 0.038 | 0.187 | 0.059 | 0.104 | 0.106 | 0.116 |
| C13.1 | 0.088 | 0.084 | 0.12 | 0.069 | 0.026 | 0.122 | 0.095 | 0.083 | 0.091 | 0.11 |
| C13.2 | 0.047 | 0.043 | 0.108 | 0.063 | 0.016 | 0.133 | 0.063 | 0.079 | 0.114 | 0.082 |
| C14.1 | 0.075 | 0.04 | 0.13 | 0.043 | 0.02 | 0.14 | 0.053 | 0.068 | 0.093 | 0.147 |
| C14.2 | 0.048 | 0.057 | 0.113 | 0.031 | 0.005 | 0.138 | 0.081 | 0.081 | 0.103 | 0.13 |
| C15.1 | 0.069 | 0.024 | 0.108 | 0.026 | 0.011 | 0.152 | 0.078 | 0.092 | 0.108 | 0.097 |
| C15.2 | 0.057 | 0.045 | 0.103 | 0.031 | 0.019 | 0.145 | 0.093 | 0.089 | 0.102 | 0.142 |
| C16.1 | 0.073 | 0.039 | 0.115 | 0.034 | 0.026 | 0.084 | 0.093 | 0.076 | 0.148 | 0.067 |
| C16.2 | 0.056 | 0.048 | 0.115 | 0.042 | 0.018 | 0.157 | 0.029 | 0.093 | 0.095 | 0.069 |
| C17.1 | 0.072 | 0.032 | 0.122 | 0.041 | 0.021 | 0.175 | 0.066 | 0.095 | 0.081 | 0.092 |
| C17.2 | 0.063 | 0.042 | 0.107 | 0.042 | 0.01 | 0.211 | 0.063 | 0.099 | 0.118 | 0.106 |
| C18.1 | 0.042 | 0.058 | 0.096 | 0.037 | 0.015 | 0.108 | 0.065 | 0.07 | 0.083 | 0.127 |
| C18.2 | 0.059 | 0.069 | 0.119 | 0.046 | 0.037 | 0.153 | 0.076 | 0.085 | 0.09 | 0.197 |
| C19.1 | 0.093 | 0.036 | 0.096 | 0.032 | 0.011 | 0.093 | 0.06 | 0.077 | 0.096 | 0.069 |
| C19.2 | 0.086 | 0.041 | 0.094 | 0.031 | 0.011 | 0.086 | 0.088 | 0.062 | 0.104 | 0.075 |
| C20.1 | 0.047 | 0.031 | 0.076 | 0.018 | 0.01 | 0.077 | 0.054 | 0.06 | 0.095 | 0.065 |
| C20.2 | 0.035 | 0.046 | 0.109 | 0.031 | 0.011 | 0.107 | 0.066 | 0.087 | 0.108 | 0.101 |
| C22.1 | 0.057 | 0.047 | 0.121 | 0.061 | 0.022 | 0.12 | 0.048 | 0.082 | 0.105 | 0.049 |
| C22.2 | 0.056 | 0.024 | 0.11 | 0.049 | 0.013 | 0.08 | 0.081 | 0.083 | 0.103 | 0.056 |
| C23.1 | 0.054 | 0.043 | 0.134 | 0.061 | 0.022 | 0.148 | 0.094 | 0.079 | 0.095 | 0.07 |
| C23.2 | 0.056 | 0.014 | 0.061 | 0.02 | 0.007 | 0.087 | 0.05 | 0.051 | 0.065 | 0.054 |
| C24.1 | 0.036 | 0.147 | 0.191 | 0.051 | 0.04 | 0.232 | 0.099 | 0.05 | 0.196 | 0.168 |
| C24.2 | 0.061 | 0.035 | 0.095 | 0.02 | 0.013 | 0.129 | 0.041 | 0.099 | 0.087 | 0.037 |
| C25.1 | 0.048 | 0.037 | 0.135 | 0.054 | 0.012 | 0.185 | 0.071 | 0.091 | 0.099 | 0.071 |
| C25.2 | 0.066 | 0.03 | 0.124 | 0.043 | 0.021 | 0.184 | 0.063 | 0.097 | 0.104 | 0.07 |
| C26.1 | 0.045 | 0.047 | 0.108 | 0.026 | 0.016 | 0.156 | 0.075 | 0.077 | 0.098 | 0.059 |
| C26.2 | 0.042 | 0.036 | 0.126 | 0.041 | 0.022 | 0.153 | 0.069 | 0.071 | 0.094 | 0.045 |
| C27.1 | 0.04 | 0.041 | 0.106 | 0.048 | 0.016 | 0.106 | 0.068 | 0.084 | 0.093 | 0.074 |
| C27.2 | 0.05 | 0.035 | 0.116 | 0.034 | 0.02 | 0.112 | 0.066 | 0.076 | 0.118 | 0.067 |
| C28.1 | 0.046 | 0.038 | 0.13 | 0.038 | 0.016 | 0.173 | 0.073 | 0.071 | 0.105 | 0.064 |
| C28.2 | 0.064 | 0.042 | 0.107 | 0.028 | 0.005 | 0.163 | 0.087 | 0.093 | 0.11 | 0.08 |
| C29.1 | 0.048 | 0.047 | 0.148 | 0.036 | 0.007 | 0.166 | 0.072 | 0.085 | 0.098 | 0.099 |
| C29.2 | 0.045 | 0.043 | 0.126 | 0.049 | 0.017 | 0.173 | 0.076 | 0.081 | 0.102 | 0.061 |
| C30.1 | 0.053 | 0.046 | 0.126 | 0.039 | 0.021 | 0.178 | 0.082 | 0.085 | 0.105 | 0.115 |
| C30.2 | 0.058 | 0.055 | 0.096 | 0.035 | 0.019 | 0.169 | 0.082 | 0.071 | 0.089 | 0.095 |
| C31.1 | 0.077 | 0.048 | 0.068 | 0.033 | 0.02 | 0.165 | 0.07 | 0.087 | 0.104 | 0.075 |
| C31.2 | 0.051 | 0.036 | 0.117 | 0.029 | 0.017 | 0.178 | 0.059 | 0.083 | 0.092 | 0.085 |
| C32.1 | 0.056 | 0.045 | 0.11 | 0.03 | 0.015 | 0.122 | 0.05 | 0.111 | 0.085 | 0.078 |
| C32.2 | 0.074 | 0.081 | 0.106 | 0.036 | 0.036 | 0.135 | 0.051 | 0.095 | 0.089 | 0.058 |

**Supplementary Table 1. DNA methylation values at ten CpG probes use to check for maternal blood contamination.** Codes in the left column designate each twin pair and each twin subject within each pair. Please note samples from twin pairs 11 and 21 have not been included in the table since they were excluded from further analysis due to either (i) dizygosity or (ii) lack of biological material available.
